# Pixelated bifunctional metasurface-driven dynamic vectorial holographic color prints for photonic security platform

**DOI:** 10.1038/s41467-021-23814-5

**Published:** 2021-06-14

**Authors:** Inki Kim, Jaehyuck Jang, Gyeongtae Kim, Jihae Lee, Trevon Badloe, Jungho Mun, Junsuk Rho

**Affiliations:** 1 Department of Mechanical Engineering, Pohang University of Science and Technology (POSTECH), Pohang, Republic of Korea; 2 Department of Chemical Engineering, Pohang University of Science and Technology (POSTECH), Pohang, Republic of Korea; 3 National Institute of Nanomaterials Technology (NINT), Pohang, Republic of Korea

**Keywords:** Nanophotonics and plasmonics, Metamaterials, Displays, Optical sensors

## Abstract

Vectorial holography has gained a lot of attention due to the promise of versatile polarization control of structured light for enhanced optical security and multi-channel optical communication. Here, we propose a bifunctional metasurface which combines both structural color printing and vectorial holography with eight polarization channels towards advanced encryption applications. The structural colour prints are observed under white light while the polarization encoded holograms are reconstructed under laser illumination. To encode multiple holographic images for different polarization states, a pixelated metasurface is adopted. As a proof-of-concept, we devise an electrically tunable optical security platform incorporated with liquid crystals. The optical security platform is doubly encrypted: an image under white light is decrypted to provide the first key and the corresponding information is used to fully unlock the encrypted information via projected vectorial holographic images. Such an electrically tunable optical security platform may enable smart labels for security and anticounterfeiting applications.

## Abstract (web summary)

The authors present a bi-functional metasurface, combining structural color printing observed under white light and polarization encoded It is appropriate. vectorial holography. A pixelated design is used encode multiple holographic images, and they demonstrate an electrically tunable optical security platform.

## Introduction

Electromagnetic waves can convey a variety of information by utilizing their distinct properties, i.e., the amplitude, phase, polarization, and orbital angular momentum (OAM) states. The characteristics of light have been utilized in diverse fields frequently seen in everyday life for example, full color displays have been realized through spectral engineering, holographic displays through outgoing light phase modulation, advanced microscopy technologies through polarization control, and optical fiber communications transmitting OAM states have all been used to take advantage of the many degrees of freedoms of light to convey a variety of information.

Metasurfaces composed of specifically designed sub-wavelength structures have received a lot of attention to freely modulate the various degrees of freedoms of light at the sub-wavelength scale. As new physical insights into light matter interactions at the nanoscale have been discovered, novel ultra-thin flat optical devices have been developed^1,2^. Some examples include color filters that realize structural color using sub-wavelength nanostructures^3,4^, and high-resolution holographic displays that exploit the geometric phase (or propagation phase) of nanostructures^5–9^ replacing conventional bulky spatial light modulators (SLM). Also, new methods of controlling polarization^10–13^ or OAM states^14–19^ of light with optical metasurfaces have been introduced, which previously required bulky wave plates or Q-plates components.

An added benefit of metasurfaces, compared to existing bulk optical systems, is that they can realize multi-functionality within a single optical element, in which multiple degrees of freedoms of light can be modulated simultaneously. For example, the conventional SLM used to manipulate a wavefront or generate holographic images can modulate only either the phase or amplitude, so is difficult to realize full space beam modulation and realistic three-dimensional (3D) holographic images. However, in the case of metasurfaces, since complex modulation that simultaneously adjusts the amplitude and phase of light is possible, full space beam modulation or realistic 3D holographic images can be realized^20–23^. In addition, dual-display devices that combine color printing and holography are also being actively studied^24–27^. Such devices show a colorful image when illuminated with white light, and an encoded holographic image is reproduced when illuminated by a laser. In other words, multiple pieces of information can be encoded into a single optical device. Thus, multifunctional metasurfaces not only enable miniaturized systems that can outperform existing optical devices, but they also open up applications in fields such as optical cryptography^28–32^ or light detection and ranging (LiDAR)^20,21,33–35^.

Vectorial holography has gained a lot of attention due to the promise of versatile polarization control of structured light for enhanced optical security and multi-channel optical communication. Previous approaches with reflective plasmonic metasurfaces to produce arbitrary polarization in the far-field suffered from low conversion efficiencies, and limited numbers of available polarization states^36^. The first vectorial meta-holography was demonstrated using di-atomic plasmonic unit cells with different distances and orientation angles to control the phase and versatile polarization states simultaneously, but was only efficient at oblique angle illumination^37^. Metasurfaces using dielectrics to produce more efficient vectorial metaholograms have been proposed, however, due to the need to exploit both the propagation and geometric phase simultaneously, the operating bandwidth was limited^38,39^. Recently a pixelated metasurface with gallium nitride (GaN) nanobricks that enables more efficient arbitrary polarization control over a broadband bandwidth has been demonstrated^11^. Moreover, to realize broadband vectorial holography by suppressing the dispersive characteristics of metasurfaces, a superposition method has been introduced, where a set of phase gradient metasurfaces relates the output polarization only to the rotation angle of the meta-atoms, independent of wavelength. Also, a metasurface doublet structure has been demonstrated to enable nondispersive white-light holographic image projection^40^. An emerging concept of arbitrary polarization conversion dichroism has been able to make a metasurface-polarizer system, that generates an arbitrarily polarized beam for unpolarized incident light^41^. In addition, the concept of orthogonal polarization dichroism has been utilized to enable independent amplitude control over arbitrary orthogonal states of polarization^42^. Such novel dichroism mechanisms are able to not only expand the degree of freedom in vectorial holography design, but also help to realize complex-amplitude vectorial holographic displays.

**Fig. 1:**
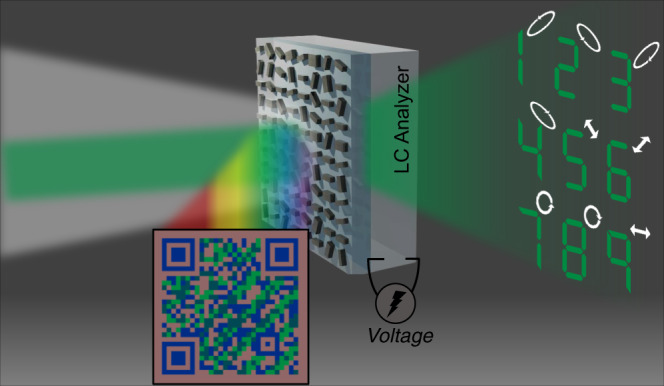
Illustration of vectorial holographic color prints using a pixelated metasurface and liquid crystal (LC) analyzer. Under ambient white light, the device displays a two-colored QR code image. On the other hand, with laser illumination the vectorial hologram images can be rendered. With an aid of LC analyzer, electrically tunable vectorial holographic color prints are realized.

Herein, we propose a bifunctional metasurface which produces a structural color print and vectorial holograms using multiple individual polarization channels, towards advanced encryption applications. The bifunctionality of the metasurface originates from the bifunctional meta-atom that acts as both a Mie-resonator and a localized half-wave plate, thus allowing the multiplexing of colors and phase at each spatial location in the metasurface. Moreover, grouping the meta-atoms allows us to assign arbitrary polarization states to the output beam. Therefore, multiple properties, i.e., the color, phase, and polarization can be engineered using our pixelated metasurface, in which encoded structural color prints can be observed under white light and fully polarized vectorial holograms can be dynamically reconstructed using a laser source in combination with an output polarizer. Previously demonstrated dual-display devices that perform color printing and holography were limited to exhibiting only two pieces of information, whereas the device proposed here is able to produce fully polarized holographic images, so that much more information can be encoded into a single device. Furthermore, in order to simplify the polarization-sorting process with a simple application of electrical bias for the fully polarized holograms, a liquid crystal (LC) modulator is used instead of the combination of a polarizer and retarder^43,44^. This electrically tunable optical security platform aims to advance two-level encryption. Firstly, the color image must be decrypted to provide a key that is used to fully unlock the encrypted information via the projected vectorial holographic images (Fig. 1).

## Results

### Design principle for the independent modulation of the phase and amplitude of light

**Fig. 2:**
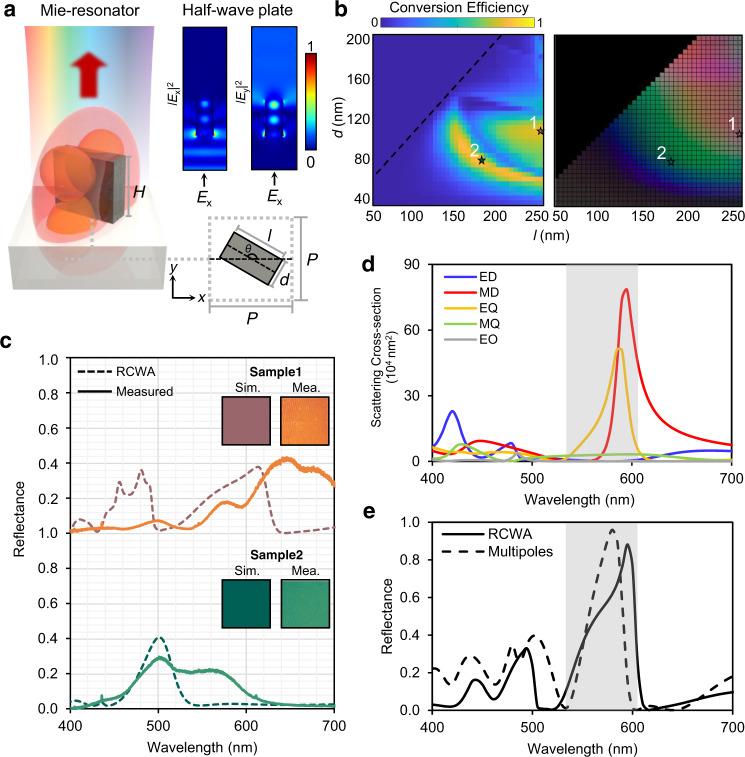
Simultaneous manipulation of reflection spectrum and phase using asymmetric meta-atoms. **a** Schematic of the multifunctional meta-atom which acts as a Mie-resonator and a localized half-wave plate. The meta-atom has length *l*, height *H*, width *d*, pitch *P*, and an in-plane rotation angle of *θ*. **b** Cross-polarization conversion efficiency at 532 nm and color palette of meta-atoms with *l* from 50 to 250 nm and *d* from 40 to 200 nm, with *H* = 350 nm and *P* = 300 nm. **c** Reflection spectra of Samples 1 and 2. Dashed line: RCWA simulation; solid line: experimental measurement. Insets represent the calculated and measured colors of Samples 1 and 2. **d** Multipole decomposition of the scattering response of the meta-atom array in free space under linearly polarized light along the *x*-axis. The meta-atoms have *l* = 250 nm, *d* = 95 nm, *H* = 350 nm, and *P* = 300 nm. Gray colored area indicates the regime where the interplay between the strong MD and EQ modes occurs. **e** Comparison between the reflectance retrieved from the multipole moments and RCWA simulation.

Metasurfaces consist of arrays of carefully designed meta-atoms. Each meta-atom can be considered as either a nanowaveguide to modulate the phase or a Mie-scatterer which resonantly scatters the incident light (Fig. 2a). In this work, we use an asymmetric bar-shaped nanowaveguide which provides two phase modulation principles, i.e., propagation phase and geometric phase. When light is incident on an asymmetric nanowaveguide, the output electric field becomes

1 $$\frac{{T}_{{\rm{L}}}+{T}_{{\rm{S}}}}{2}\left[\begin{array}{c}1\\ \mp {\rm{i}}\end{array}\right]+\frac{{T}_{{\rm{L}}}-{T}_{{\rm{S}}}}{2}{e}^{\pm i2\varphi (x,y)}\left[\begin{array}{c}1\\ \pm {\rm{i}}\end{array}\right],$$

where ${T}_{{\rm{L}}}$ and ${T}_{{\rm{S}}}$ represent the complex transmission coefficients along the long and short dimensions of the anisotropic nanowaveguide, and $\varphi {\boldsymbol{(}}x{\boldsymbol{,}}y{\boldsymbol{)}}$ is the in-plane rotation angle of the nanowaveguide. The terms $\frac{{T}_{{\rm{L}}}+{T}_{{\rm{S}}}}{2}$ and $\frac{{T}_{{\rm{L}}}-{T}_{{\rm{S}}}}{2}$ are related to propagation phase, which depends on the geometry of the waveguide (independent of the in-plane rotation angle). The ${e}^{\pm i2\varphi (x,y)}$ term represents the geometric phase, which describes the extra phase added due to the in-plane rotation of the meta-atom (independent of the geometry). Therefore, the phase of co-polarized and cross-polarized light passing through the meta-atom can be modulated by changing its geometry and rotation angle. For instance, asymmetric silicon (Si) nanopillars can flip the polarization vector of incident wave, thus acting as a half-wave plate with the optical axis. The polarization conversion efficiency of Si nanopillars with height *H* = 350 nm, pixel pitch *P* = 300 nm, length *l* from 50 to 250 nm, and width *d* from 40 to 200 nm was calculated using the rigorous coupled wave analysis (RCWA) method (Fig. 2a, b—Left).

The constituent meta-atoms of a metasurface can also be understood as radiating multipole sources, i.e., Mie-scatterers. Mie-scatterers resonantly radiate incident light when the wavelength is comparable to the effective size of the scatterer. Thus, the scattering response greatly differs based on the geometry and refractive index of the scatterer. For example, Si nanopillars with different geometries have distinct reflection spectra in the visible regime which can be seen as distinct colors (Fig. 2b—Right and Fig. 2c). The fundamentals of spectral modulation using high-index Mie-scatterers are mostly related to interference between radiating multipoles, such as the electric dipole (ED) and magnetic dipole (MD)^3,32^. The far-field interference between these multipoles can be understood using the multipole moments calculated by multipole expansion techniques, for instance, the exact Cartesian multipole (a more detailed explanation is given in the “Methods” section); these retrieved multipole moments are used to successfully calculate the reflection and transmission coefficients of the whole metasurface^45^. As an example, the scattering response of an array of nanopillars with *H* = 350 nm, *P* = 300 nm, *l* = 250 nm, and *d* = 95 nm was analyzed using multipole decomposition (Fig. 2d). Unlike conventional high-index Huygens sources, excitation of electric quadrupole (EQ), magnetic quadrupole (MQ), and electric octupole (EO) modes are observed; the high aspect ratio required for the meta-atoms to have both proper reflectance and polarization conversion efficiency leads to the inevitable excitation of the higher modes. In the high-frequency regime, most multipoles have comparable amplitudes so that complex far-field interactions are expected. However, no responses such as directional scattering are observed due to the losses in the Si (Supplementary Note 1 and Supplementary Fig. 1). The most interesting regime is from around 540 nm to 620 nm where the EQ, MD, and MQ are dominant (Fig. 2d—colored in gray), and the optical losses are low. The interplay between the multipole modes results in near-zero reflectance at the boundaries of the regime and maximized reflectance in the center (Fig. 2e). Such far-field responses originate from the interference of the multipole family. This is underpinned by the fact that the retrieved reflection spectrum made of multipole moments closely matches with numerically calculated one using RCWA simulations (Fig. 2e). The differences between the two results may come from the incomplete equations derived with the assumption that other possibly excited orders are negligible. Deriving the exact equations that include all possible orders could be an interesting topic for further investigation, but it is beyond the scope of this work. In conclusion, our nanopillars act as Mie-resonators, and their far-field responses are modulated based on the excited multipole moments inside the structure.

The two different points-of-view of the meta-atom acting as a waveguide and a Mie-scatterer are not independent; which point of view is more appropriate is often determined by the height-to-width ratio of the meta-atom^46,47^. However, a recent study showed that the intermediate range where the two perspectives can both be applied might exist^24^. In other words, the meta-atom can simultaneously act as a waveguide and a Mie-scatterer, which implies the capability of the meta-atom to independently modulate phase (waveguide) and reflection spectra (Mie-scatterer) to some degree. This simultaneous modulation would lead to bifunctional metasurfaces that integrate structural coloring and holographic imaging into a single device. In this study, the intermediate range between the two different points-of-view is explored by analyzing Si meta-atom with RCWA and multipole decomposition (Fig. 2b–d). As an example, two Si nanopillars (Sample 1, 2 and Fig. 2b-c) which have the same polarization conversion efficiency, but distinct spectral responses are prepared. The nanopillars work as a half-wave plate with the same efficiency (Fig. 2b—Left), but produce distinct far-field radiation from Mie-scattering (Fig. 2c, e). The distinct spectra are achieved by adjusting the geometric parameters (i.e., length *l* and width *d*), thus rendering different structural colors. The reflection spectra in Sample 1 and 2 from RCWA simulations and measurements show a little discrepancy, which arises from the clockwise and anticlockwise rotation of the nanostructure group in the samples prepared for both reflection and conversion efficiency measurements (Supplementary Note 2 and Supplementary Fig. 2). In terms of holographic storage, the phase is encoded by rotating the angle of the nanopillars, i.e., through geometric phase. The important thing to note is that the two nanopillars have the same conversion efficiency ${\left|\frac{{T}_{{\rm{L}}}-{T}_{{\rm{S}}}}{2}\right|}^{2}$, but a different complex number $\frac{{T}_{{\rm{L}}}-{T}_{{\rm{S}}}}{2}$. Therefore, the phase part of the $\frac{{T}_{{\rm{L}}}-{T}_{{\rm{S}}}}{2}$ term is the kind of retardation or propagation phase *α*(*x, y*) that must also be compensated for when realizing the phase of the hologram. Considering the propagation phase term, the resultant phase retarded by the two nanopillars are ${\alpha }_{1}\left(x,y\right)\pm 2\varphi \left(x,y\right)$ and ${\alpha }_{2}\left(x,y\right)\pm 2\varphi \left(x,y\right)$, respectively, where the + sign represents right-handed circularly polarized light (RCP) and the – sign left-handed circularly polarized light (LCP). The propagation phase difference ${\alpha }_{2}\left(x,y\right)-{\alpha }_{1}(x,y)$ of the two nanopillars with different geometric parameters, should be compensated in advance with an additional rotation angle of $\frac{{\alpha }_{2}\left(x,y\right)-{\alpha }_{1}(x,y)}{2}$ (Supplementary Note 3 and Supplementary Fig. 3) resulting in an additional geometric phase ${\alpha }_{2}\left(x,y\right)-{\alpha }_{1}(x,y)$.

Furthermore, in order to minimize the absorption of Si in the high frequency visible regime and to increase device efficiency (i.e., to improve the color generation and high hologram efficiency), hydrogenated amorphous silicon (a-Si:H) is deposited using plasma-enhanced chemical vapor deposition at an optimized pressure and temperature, 25 mTorr and 200 °C, respectively (Supplementary Note 4 and Supplementary Fig. 4)^48^.The used a-Si:H is denoted as Si(P25) and exhibits much lower extinction coefficient at the wavelength of 532 nm with enough refractive index compared to conventional a-Si, resulting in improved device efficiency (Fig. 2b—Left).

### Vectorial holography-based multiplexing

**Fig. 3:**
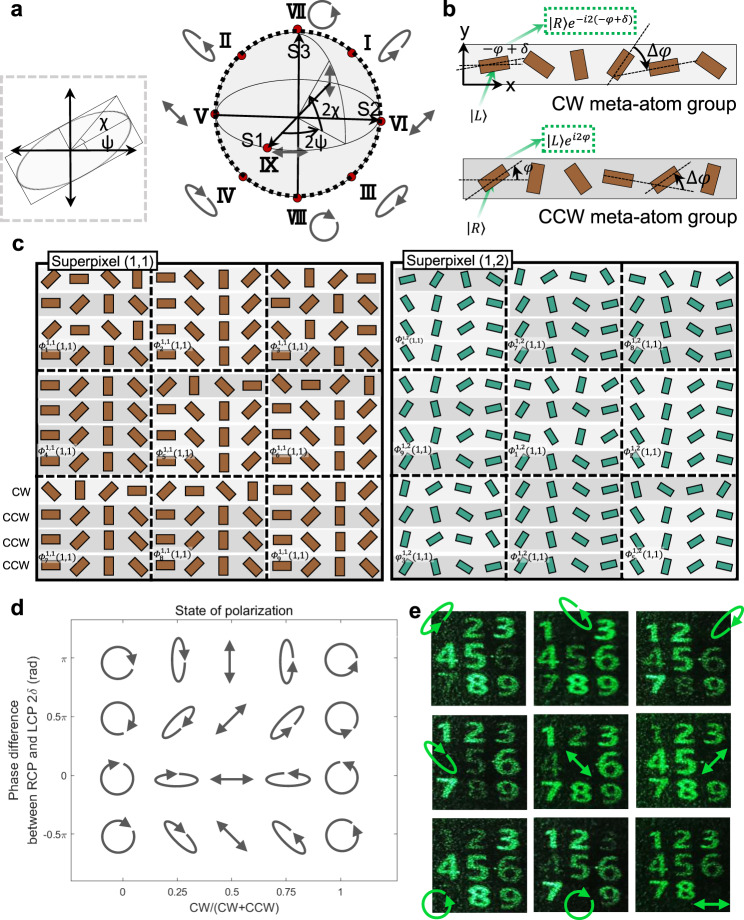
Design method of vectorial hologram using PB phase-based pixelated metasurfaces. **a** Polarization ellipse and Poincare sphere representing polarization states with different azimuth *ψ* and ellipticity *χ* angles. Polarization states I to IX are experimentally demonstrated. **b** Two types of meta-atom groups to generate outgoing RCP and LCP with arbitrary phase. **c** Schematic of the metasurfaces that are composed of two different sized meta-atoms exhibiting color prints and pixelated for vectorial holographic devices. The initial phase difference between two different sized meta-atoms is compensated and the subpixels at each superpixel are randomly ordered to remove the unwanted grating effect. **d** Realization of the arbitrary polarization states by changing the relative phase and amplitude between RCP and LCP. On the Poincaré sphere, the longitudinal and latitudinal variation is modulated by arbitrary phase and amplitude of two circular polarizations. **e** Experimental demonstration of the hologram images carrying nine different polarization states. Each holographic image is blocked by the corresponding combination of wave retarder and polarizer.

Vectorial holographic devices are able to produce holographic images with spatially varying polarization states. In contrast to a conventional hologram that possesses a uniform polarization state, a vectorial hologram has more degrees of freedom, proportional to the number of encoded polarization states. The polarization state is defined by the azimuth *ψ* and ellipticity *χ* angles (Fig. 3a—Left). Each polarization state with two degrees of freedom can be represented by a point (*r*, 2*ψ*, 2*χ*) on the surface of Poincaré sphere, where *r* denotes the intensity of the light with fixed value (Fig. 3a—Right). For example, every linear polarization state is represented by a point on the surface of S1–S2 plane. RCP and LCP are located on the north and south pole of the S3 axis, and the intermediate right and left elliptical polarizations are located on the northern and southern hemisphere, respectively. Any polarization state can be constructed by a superposition of the RCP and LCP with an arbitrary phase and amplitude. The azimuth *ψ* and ellipticity *χ* angles are described as

2 $$2{\psi} =2{\delta}\,{\mathrm{and}}\,2{\chi} ={{{\mathrm{sin}}}^{-1}}\frac{{{a}_{\mathrm{R}}}^{2}\,-\,{{a}_{\mathrm{L}}}^{2}}{{{a}_{{\mathrm{R}}}^{2}\,+\,{{a}_{\mathrm{L}}}}^{2}},$$

where $2\delta$ is the phase difference between RCP and LCP, and ${a}_{{\rm{R}}}$ and ${a}_{{\rm{L}}}$ are the amplitude of RCP and LCP, respectively. The full azimuth angle 2*ψ* can be achieved by modulating the phase difference $2\delta$ between RCP and LCP from 0 to 2π, resulting in every linear polarization state on the S1-S2 plane. The other states on the northern and southern hemisphere of the Poincaré sphere can be realized by modulating the amplitude of RCP and LCP (${a}_{{\rm{R}}}$ and ${a}_{{\rm{L}}}$) from 0 to 1, covering the full ellipticity angle. As a result, the RCP and LCP with an intentionally selected phase and amplitude can generate any polarization states.

The phase-gradient metasurface is adopted to achieve the required circular polarizations (CPs). Using geometric phase, the meta-atoms with rotation angle $\varphi (x,y)$ can impose $2\varphi {\boldsymbol{(}}x{\boldsymbol{,}}y{\boldsymbol{)}}$ $(-2\varphi (x,y))$ phase retardation for RCP (LCP) incidence, where the rotation angle is measured in counterclockwise (CCW) direction (Fig. 3b). For the meta-atom groups composed of meta-atoms whose angle increment satisfies $\triangle \varphi\, > \, 0$ (Fig. 3b—CCW group), the phase-gradient $\frac{2\triangle \varphi }{P}$ along the *x* axis is obtained at the outgoing LCP, where *P* denotes the pixel pitch. As a result, according to the generalized Snell’s law, the outgoing LCP is deflected to the angle ${\theta}_{{\mathrm{d}}}={\mathrm{arcsin}}(\frac{2{\triangle} {\varphi} }{{k}_{0}P})$, where ${k}_{0}$ denotes the free space wavenumber^49^. Likewise, the same amount of phase-gradient and deflection angle at the outgoing RCP is achieved by the meta-atom groups satisfying $\triangle \varphi\, <\, 0$ (Fig. 3b—clockwise (CW) group). On the premise that both RCP and LCP are deflected to same angle by modulating relative angle increment $\triangle \varphi$, the phase of the outgoing RCP and LCP is modulated by the absolute angle of the meta-atom: $\varphi$ and $-\varphi +\delta$ as shown in Fig. 3b that result in 2*δ* difference between CPs. Then, the amplitude of the RCP and LCP in transmitted wave is achieved by modulating the number of CW and CCW groups. We design a superpixel to produce multiple polarization states in single device as shown in Fig. 3c. Each superpixel consists of nine types of subpixel, generating nine polarization states. In other words, multiple polarization states can be realized at corresponding subpixels with different combination of phase and amplitude difference between CPs (Fig. 3d). The number of meta-atoms that make up the CW and CCW groups are determined considering the deflection efficiency (Supplementary Note 5 and Supplementary Fig. 5). Moreover, a twin-image is inherently generated from metasurfaces composed of these two phase-gradient groups (CW and CCW) illuminated by linearly polarized light. The effect of the twin-image on the total diffraction efficiency is numerically analyzed and experimentally characterized in Supplementary Note 6 and Supplementary Fig. 6.

To reconstruct the holographic images carrying different polarization states, the phase distribution of the hologram should be encoded, where the iterative Gerchberg–Saxton (GS) algorithm was used to retrieve the phase distribution. The phase at each position is denoted as ${\varPhi }_{{\rm{n}}}^{{\rm{X,Y}}}\left(x,y\right)$, as shown in Fig. 3c, where *n* is the types of polarization state, (*x, y*) denotes the position of the meta-atoms within the subpixel, and (*X, Y*) denotes the position of the superpixel within the entire metasurface. When the ${\varPhi }_{{\rm{n}}}^{{\rm{X,Y}}}\left({\mathrm{1,1}}\right)$ is exploited to encode the phase of the hologram, remaining pixels except ${\varPhi }_{{\rm{n}}}^{{\rm{X,Y}}}\left({\mathrm{1,1}}\right)$ in each subpixel are determined by the design principle of realizing arbitrary polarization states. It is noted that the subpixels are randomly placed within each superpixel to eliminate the grating effect induced from pixelated metasurface^11^. As a proof-of-concept, we experimentally demonstrated nine holographic images each showing a single number from 1 to 9, as shown in Fig. 3e, and the polarization of each number corresponds to the locations I to IX denoted on the Poincaré sphere, respectively. The feasibility of the encoded polarization states is proved by using appropriate analyzer blocking the orthogonal polarization states. When the one of the polarization states is blocked, the intensities of the others are also reduced. This is described in more detail using Jones matrices in Supplementary Note 7 with a comparison of the experimental and simulated results (Supplementary Fig. 7).

### Demonstration of liquid crystal-assisted vectorial holographic color prints

**Fig. 4:**
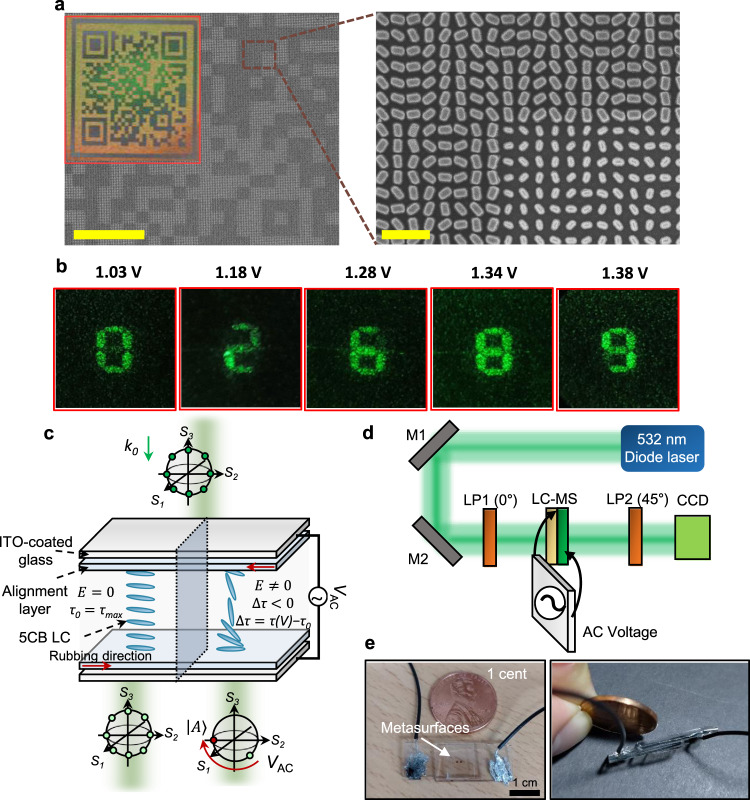
Experimental demonstration of electrically tunable vectorial holographic device. **a** Optical (inset) and scanning electron microscope images of the fabricated device. Scale bars represent 50 µm and 1 µm, respectively. **b** Electrically tunable vectorial metaholograms according to different applied voltages on liquid crystals (LCs). **c** LC cell design schematic. Initial LC molecules are ordered parallel to the rubbing direction of polyimide alignment layer, where retardation $\tau$ of transmitted light has maximum value. By applying a voltage, the LC is ordered parallel to the electric bias direction, decreasing the retardation resulting in polarization change of transmitted light. All of the polarization states are tuned to be 135° linearly polarized light. **d** Optical setup schematic for vectorial hologram polarization-sorting process. (M1 mirror 1, M2 mirror 2, LP1 linear polarizer 1, LC-MS LC-integrated metasurfaces, LP2 linear polarizer, CCD charge coupled device) The 135° polarized light outgoing from the LC cell is blocked at LP2 and the corresponding holographic images are captured with a CCD. **e** Photographs of the fabricated device.

To implement a straightforward and electrically tunable sorting out-process of vectorial holographic images, an LC cell is incorporated onto the fabricated metasurface (Fig. 4a). Particularly, for dynamic vectorial holographic color print applications, we fabricate another device. Five different sections of the holographic number are assigned to five different polarization states. By applying a voltage to the LC cell, the pre-assigned polarization states can be controlled and sorted out in sequence in the same position (Fig. 4b–d). The LC orientation changes induce an effective refractive index (∆*n*_eff_) shift; $\triangle {n}_{{\rm{eff}}}=({n}_{{\rm{o}}}{n}_{{\rm{e}}}/\sqrt{{n}_{{\rm{o}}}^{2}{{\cos }}^{2}\theta +{n}_{{\rm{e}}}^{2}{{\sin }}^{2}\theta })-{n}_{{\rm{o}}},$ where *n*_e_ and *n*_o_ are the extraordinary and ordinary reflective index of the LCs, respectively, and *θ* = 0° represents the tangential orientation with respect to the rubbing direction. As a result, the LC cell can be used as a phase retarder where the phase retardation *τ* [rad] can be determined as $\tau ={\int _{0}^{{\rm{t}}}}2\pi \triangle {n}_{{\rm{eff}}}(z)/\lambda {{\rm{d}}z},$ where *t* is the thickness of the LC cell, and *λ* is the wavelength of the transmitted light^43,44^. The LC-integrated metasurface device is designed as to exhibit a specific polarization changing trajectory, when the light propagates through the LC medium (Fig. 4c, e). A detailed explanation of the LC cell design method can be found in Supplementary Note 8 and Supplementary Fig. 8. The trajectory is represented as a continuous path on the surface of S2–S3 plane in the Poincaré sphere, denoted by a dotted line in Fig. 3a. As a proof of concept, we implement a vectorial hologram encoded to produce numerical digits for five different polarization states, which are +45° linear polarization, −45° linear polarization, RCP, and LCP on the S2–S3 plane surface, and linearly horizontal polarized light (LHP) not on the surface which is not affected by the LC medium (because the slow axis of the LC medium is parallel to LHP). The four polarization states on the trajectory are evolved around the S1 axis by applying different voltages, but the LHP located on the axis of the surface does not change regardless of the change in voltage. The four different polarization states are modulated to have 135° linear polarization states by an appropriate electric bias. By attaching a 45° linear polarizer behind the LC, it was experimentally confirmed that the four polarization components can be turned off at the corresponding voltages (Fig. 4b–d). Such a strategy enables real-time video holographic displays with a single flat-optical device that does not demand a further external light modulator (e.g., SLM) or optical components.

**Fig. 5:**
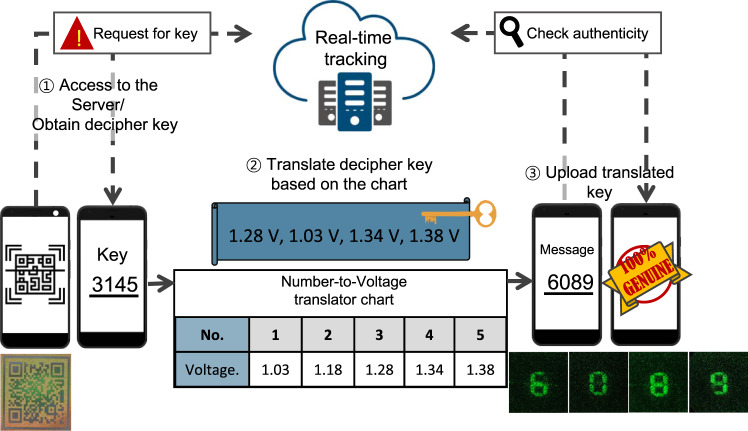
Vectorial holographic color prints based two-level security platform. Firstly, a user who wants to unlock the system should scan the smart label exhibiting reflection image of two-colored QR code using smart phone or relevant scanners. Such access information is real-time tracked at the main server and the first key will be distributed. Secondly, the first key will be translated to inform the user of the voltage value which will be applied on our LC-integrated device. Then, automatically the smart label will produce holographic images in a space, which is the second key to unlock the system. At the same time, such access will also be transmitted into the main server to finally check the authenticity (See Supplementary Video 1).

Furthermore, we propose a two-level optical security platform. Our LC-assisted vectorial hologram operates in both reflection and transmission mode simultaneously. The two colored QR codes can be seen with the naked eye under ambient white light (or to magnify the printed image, an optical microscope can be used). At the same time, under illumination of linearly polarized light, fully polarized vectorial holographic images can be produced in the far field. Using such a bi-functional metasurface, two independent pieces of information can be encoded into a single device, which is promising for an anti-counterfeiting platform with enhanced security. The first-level key is linked to the QR code that can be decrypted through a camera. At this stage, a specific set of random numbers will be given to the user. This could be achieved through a main server that detects the access information and distributes the random numbers. These numbers are then translated to the second-level key (voltage values) using a predefined chart. Then, the decrypted voltage information from the chart can be applied to the LC in sequence and by selectively turning off the polarized images, four different holographic digits can be produced in a far-field image plane (Fig. 5) (Supplementary Video 1). The final decrypted password information from the electrically tunable hologram is used as a real-time tracking platform to check authenticity (Fig. 5). Moreover, we would like to highlight the usefulness of the structural coloration for the first key distribution. Multi-dimensional QR codes that possess higher security levels with multiple pieces of encoded information can be generated by merging multiple QR codes^50^. It should be noted that four-color information is required to merge two independent QR codes, due to the decoding process that requires the color information to split the two codes. With our design principle of multiplexing colorprints and vectorial holograms, the density of information can be increased by merging QR codes with four different sized nanostructures while maintaining the performance of far-field holographic images. We experimentally realized a multidimensional QR code which was generated by merging two independent QR codes and far-field vectorial holographic images (Supplementary Note 9 and Supplementary Fig. 9). Such systems could be implemented into high security smart labels with a single-layered device without the aid of additional mechanical or electronic devices. We also anticipate that the entire system could be further miniaturized by directly integrating the illuminating light source with the metasurface on a single chip using a vertical cavity surface emitting laser^34^.

## Discussion

In conclusion, we have numerically and experimentally demonstrated vectorial holographic color prints which have the multifunctionality of structural color prints and LC-assisted dynamic vectorial holographic image projection. By exploiting multiple properties of light (e.g., the colors, phase, and polarization), optical information channels have been drastically increased promising for advanced optical encryption platform. To simultaneously modulate the required phase at each pixel and the reflection spectrum, two types of nanopillars with different geometrical parameters (length and width) were used, and the propagation phase difference of each nanopillar was compensated for by an additional rotation of the nanopillar (to impose additional geometric phase). In addition, we simplified the polarization-sorting process with an electrically tunable LC modulator for dynamic vectorial holograms. Such holographic video displays can be utilized as two-level security applications or internet-of-things sensors. We envision that the proposed ultra-compact vectorial holographic color prints could be a vital anticounterfeiting platform, which can be directly printed onto valuable products as a form of security smart label.

## Methods

### Full-wave 3D simulation

An in-house developed RCWA algorithm was used to calculate the reflection and transmission spectra of the nanostructures. The spectra were converted to chromatic information by using the MATLAB toolbox (Optprop toolbox by Jerker Wågberg (https://www.mathworks.com/matlabcentral/fileexchange/13788-optprop-a-color-properties-toolbox), MATLAB Central File Exchange. Retrieved September 1, 2020). The D50 illumination source with the CIE standard observer was used throughout.

### Multipole decomposition

COMSOL Multiphysics, a commercial finite-element method (FEM) software, was used to calculate the decomposed multipole scattering cross-sections. The exact Cartesian multipole moments up to octupole were calculated using the equations in ref. ^51^.

### Metasurface fabrication

Metasurfaces were fabricated on a glass substrate. A 350 nm-thick layer of a-Si:H was deposited using plasma enhanced chemical vapor deposition (PE-CVD, BMR Technology HiDep-SC) with a flow rate of 10 sccm for silane (SiH_4_) and 75 sccm hydrogen (H_2_) gases at the optimized pressure and temperature, 25 mTorr and 200 °C. Metasurface patterns were transferred onto the positive tone photoresist (polymethyl methacrylate, PMMA, Microchem) using a standard electron beam lithography process (ELIONIX, ELS-7800, accelerating voltage of 80 kV, beam current of 100 pA). After the development process, a 50 nm-thick chromium (Cr) layer was deposited and lift-offed. The Cr etch mask was used to transfer the metasurface pattern onto the a-Si:H layer using a dry etching process (DMS, silicon/metal hybrid etcher). Finally, the Cr etch mask was removed using Cr etchant (CR-7).

### LC cell fabrication

The LC cell operating as polarization modulator was fabricated on glass plates coated with indium tin oxide (ITO) and polyimides (Nissan Chemical Korea) alignment layers. The polyimides were spin-coated at 1000 rpm for 10 s and 2500 rpm for 30 s. The spin-coated layer was baked at 230 °C for 60 min. The polyimides layer was rubbed to form a unidirectional alignment of LCs. Two ITO-glasses prepared by such process were assembled using a mixture of glass spacer and UV-glue (Norland Products Inc., NOA 65) to form a 10 µm gap.

### Measurement of reflection spectrum and optical image

A homemade optical setup was built to measure the reflection spectra. The light source was a Xenon lamp (Newport). The reflected power was measured using a spectrometer (HORIBA, iHR320) with a 20× (NA = 0.4) lens (Olympus, LMPlanFL N). The optical image was captured using a microscope (Olympus, MX63) with a halogen lamp (Olympus, TH4-200) and a 20× (NA = 0.4) objective.

## Supplementary information

Supplementary Information

Description of Additional Supplementary Files

Supplementary Movie 1

## Data Availability

The data that support the findings of this study are available from the corresponding author upon reasonable request.

## Code availability

The code used for the meta-hologram design is available from the corresponding author upon reasonable request.

## References

[1] Kim I (2018). Outfitting next generation display with optical metasurfaces. ACS Photon..

[2] Jeong H (2020). Emerging advanced metasurfaces: alternatives to conventional bulk optical devices. Microeletron. Eng..

[3] Jang J (2020). Spectral modulation through the hybridization of Mie-scatterers and quasi-guided mode resonances: realizing full and gradients of structural color. ACS Nano.

[4] Joo W-J (2020). Metasurface-driven OLED displays beyond 10,000 pixels per inch. Science.

[5] Zheng G (2015). Metasurface holograms reaching 80% efficiency. Nat. Nanotechnol..

[6] Mueller JPB, Rubin NA, Devlin RC, Groever B, Capasso F (2017). Metasurface polarization optics: Independent phase control of arbitrary orthogonal states of polarization. Phys. Rev. Lett..

[7] Li Z (2017). Dielectric meta-holograms enabled with dual magnetic resonances in visible light. ACS Nano.

[8] Ansari MA (2019). A spin-encoded all-dielectric metahologram for visible light. Laser Photon. Rev..

[9] Ansari MA (2020). Engineering spin and antiferromagnetic resonances to realize an efficient direction-multiplexed visible meta-hologram. Nanoscale Horiz..

[10] Zang X (2018). Polarization encoded color image embedded in a dielectric metasurface. Adv. Mater..

[11] Song Q (2020). Ptychography retrieval of fully polarized holograms from geometric-phase metasurfaces. Nat. Commun..

[12] Intaravanne Y (2021). Phase manipulation based polarization profile realization and hybrid holograms using geometric metasurface. Adv. Photon. Res..

[13] Intaravanne Y, Chen X (2020). Recent advances in optical metasurfaces for polarization detection and engineered polarization profiles. Nanophotonics.

[14] Ren H (2020). Complex-amplitude metasurface-based orbital angular momentum holography in momentum space. Nat. Nanotechnol..

[15] Fang X, Ren H, Gu M (2020). Orbital angular momentum holography for high-security encryption. Nat. Photon..

[16] Ren H (2019). Metasurface orbital angular momentum holography. Nat. Commun..

[17] Jin L (2019). Dielectric multi-momentum meta-transformer in the visible. Nat. Commun..

[18] Mahmood N (2019). Twisted non-diffracting beams through all dielectric meta-axicon. Nanoscale.

[19] Mahmood N (2018). Polarisation insensitive multifunctional metasurfaces based on all-dielectric nanowaveguides. Nanoscale.

[20] Park J (2021). All-solid-state spatial light modulator with independent phase and amplitude control for three-dimensional LiDAR applications. Nat. Nanotechnol..

[21] Kim I (2021). Nanophotonics for light detection and ranging technology. Nat. Nanotechnol..

[22] Lee G-Y (2017). Complete amplitude and phase control of light using broadband holographic metasurfaces. Nanoscale.

[23] Overvig AC (2019). Dielectric metasurfaces for complete and independent control of the optical amplitude and phase. Light.

[24] Yoon G, Lee D, Nam KT, Rho J (2018). “Crypto-display” in dual-mode metasurfaces by simultaneous control of phase and spectral responses. ACS Nano.

[25] Lim KTP, Liu H, Liu Y, Yang JKW (2019). Structural color three-dimensional printing by shrinking photonic crystal. Nat. Commun..

[26] Wei Q (2019). Simultaneous spectral and spatial modulation for color printing and holography using all-dielectric metasurfaces. Nano Lett..

[27] Wen D, Cadusch JJ, Meng J, Crozier KB (2020). Multifunctional dielectric metasurfaces consisting of color holograms encoded into color printed images. Adv. Funct. Mater..

[28] Qu G (2020). Reprogrammable meta-hologram for optical encryption. Nat. Commun..

[29] Sung J, Lee G-Y, Choi C, Hong J, Lee B (2020). Polarization-dependent asymmetric transmission using a bifacial metasurfac. Nanoscale Horiz..

[30] Luo X (2020). Integrated metasurfaces with microprints and helicity-multiplexed holograms for real-time optical encryption. Adv. Opt. Mater..

[31] Xue J (2019). Perturbative countersurveillance metaoptics with compound nanosieve. Light.

[32] Jang J (2018). Kerker-conditioned dynamic cryptographic nanoprints. Adv. Opt. Mater..

[33] Li S-Q (2019). Phase-only transmissive spatial light modulator based on tunable dielectric metasurface. Science.

[34] Xie Y-Y (2020). Metasurface-integrated vertical cavity surface-emitting lasers for programmable directional lasing emissions. Nat. Nanotechnol..

[35] Li Z (2018). Full-space cloud of random points with a scrambling metasurface. Light.

[36] Wu PC (2017). Versatile polarization generation with an aluminum plasmonic metasurface. Nano Lett..

[37] Deng Z-L (2018). Diatomic metasurface for vectorial holography. Nano Lett..

[38] Zhao R (2018). Multichannel vectorial holographic display and encryption. Light.

[39] Arbabi E, Kamali SM, Arbabi A, Faraon A (2019). Vectorial holograms with a dielectric metasurface: ultimate polarization pattern generation. ACS Photon..

[40] Song Q (2021). Bandwidth-unlimited polarization-maintaining metasurfaces. Sci. Adv..

[41] Wang S (2021). Arbitrary polarization conversion dichroism metasurfaces for all-in-one full Poincaré sphere polarizers. Light.

[42] Fan, Q. et al. Independent amplitude control of arbitrary orthogonal states of polarization via dielectric metasurfaces. *Phys. Rev. Lett*. **125**, 267402 (2020). 10.1103/PhysRevLett.125.267402 PMC8143859 33449781

[43] Kim I (2020). Stimuli-responsive dynamic metaholographic displays with designer liquid crystal modulators. Adv. Mater..

[44] Kim I (2021). Holographic metasurface gas sensors for instantaneous visual alarms. Sci. Adv..

[45] Terekhov PD (2019). Multipole analysis of dielectric metasurfaces composed of nonspherical nanoparticles and lattice invisibility effect. Phys. Rev. B.

[46] Lalanne P, Chavel P (2017). Metalenses at visible wavelengths: past, present, perspectives. Laser Photon. Rev..

[47] Zhan A (2016). Low-contrast dielectric metasurface optics. ACS Photon..

[48] Yang Y (2021). Revealing structural disorder in hydrogenated amorphous silicon for a low-loss photonic platform at visible frequencies. Adv. Mater..

[49] Yu N (2011). Light propagation with phase discontinuities: generalized laws of reflection and refraction. Science.

[50] You M (2016). Three-dimensional quick response code based on inkjet printing of upconversion fluorescent nanoparticles for drug anti-counterfeiting. Nanoscale.

[51] Mun J, So S, Jang J, Rho J (2020). Describing meta-atoms using the exact higher-order polarizability tensors. ACS Photon..

