# Supplementary material for: Pixelated bifunctional metasurface-driven dynamic vectorial holographic color prints for photonic security platform
Source: Nat Commun. 2021 Jun 14;12:3614. doi: 10.1038/s41467-021-23814-5 (PMC8203667; doi:10.1038/s41467-021-23814-5)
Supplement: Supplementary file 1 — Supplementary Information [file 41467_2021_23814_MOESM1_ESM.docx]

**Supplementary Information**

**Pixelated bifunctional metasurface-driven dynamic vectorial holographic color prints for photonic security platform**

Inki Kim^1,#^, Jaehyuck Jang^2,#^, Gyeongtae Kim^1,#^, Jihae Lee^2^, Trevon Badloe^1^, Jungho Mun^2^, Junsuk Rho^1,2,3,^*

^1^Department of Mechanical Engineering, Pohang University of Science and Technology (POSTECH), Pohang 37673, Republic of Korea

^2^Department of Chemical Engineering, Pohang University of Science and Technology (POSTECH), Pohang 37673, Republic of Korea

^3^National Institute of Nanomaterials Technology (NINT), Pohang 37673, Republic of Korea

*Corresponding author. E-mail: [jsrho@postech.ac.kr](mailto:jsrho@postech.ac.kr)

^#^These authors contributed equally to this work

**Supplementary Note1. Multipole decomposition of the periodic nanopillars**

To understand the contributions of the multipole modes to the far-field responses, we implemented multipole decomposition (up to octupole) of periodic nanopillars which have *h* of 350 nm, *l* of 250 nm, and *w* of 95 nm based on the expression in ref. 1 (Supplementary Figure 1a, b). Members of multipole family are known to interfere, creating many interesting far-field responses. Accordingly, the multipole moments (up to octupole) can be used to derive reflection coefficient *r* and transmission coefficient *t* as following [2]:

|  | $r=\frac{ik_{d}}{E_{0}2P^{2}\varepsilon_{d}}\left( p_{x}-\frac{1}{v_{d}}m_{y}+\frac{ik_{d}}{6}Q_{xz}-\frac{ik_{d}}{v_{d}}M_{yz-}\frac{k_{d}^{2}}{6}O_{xzz} \right),$ | | | (1) |
| --- | --- | --- | --- | --- |
|  | $t=1+\frac{ik_{d}}{E_{0}2P^{2}\varepsilon_{d}}\left( p_{x}+\frac{1}{v_{d}}m_{y}-\frac{ik_{d}}{6}Q_{xz}-\frac{ik_{d}}{v_{d}}M_{yz-}\frac{k_{d}^{2}}{6}O_{xzz} \right),$ | | | (2) |
|  | | $R=\left\vert r \right\vert^{2},$ | (3) | |
|  | | $T=\left\vert t \right\vert^{2},$ | (4) | |

where *k*_d_: wavevector in surrounding medium; *ε*_d_: permittivity of surrounding medium; *v*_d_: speed of light in the medium; *p*_x_: *x*-component of ED moment; *m*_y_: *y*-component of MD moment; *Q*_xz_: *xz*-component of EQ moment; *M*_yz_: *yz*-component of MQ moment; *O*_xzz_: *xzz*-component of EO moment. In the high frequency regime, most multipoles have comparable amplitudes so that interesting scattering responses were expected. However, no responses such as directional scattering are observed due to the high losses in the silicon (Supplementary Figure 1c). The most interesting regime is from around 540 nm to 620 nm where EQ, MD, and MQ are dominant (colored in gray), where the optical losses are low. The interplay between the multipole modes result in near zero reflectance at the boundaries of the regime, and maximized reflectance in the middle (Supplementary Figure 1c). Such far-field responses originate from the interference of the multipole family, underpinned by the retrieved reflection (Supplementary Figure 1d) based on equations (1) to (4). We compared the retrieved reflection from the multipole moments using equation (1) and (3) and numerically calculated reflection using RCWA simulations (Supplementary Figure 1d). Both results show good agreement in the resonant peaks. The differences between the two results may come from the incomplete equations derived with the assumption that other possibly excited orders, for example, *Q*_xx_, *Q*_yz_, *Q*_xy_, *Q*_yy_, *Q*_zz_ are negligible. Deriving the exact equations that include all possible orders could be an interesting topic for further work, but it is beyond the scope of this work.

**Supplementary Figure 1. Multipole decomposition and numerical analysis of the periodic nanopillars.** (a) Scattering cross-section up to quadrupole calculated based on exact Cartesian multipoles. Blue mark: ED; Red mark: MD; Yellow mark: EQ; Green mark: MQ; Grey mark: EO. (b) Phase of the multipole moments. (c) Reflectance, transmittance, and absorption calculated using RCWA simulation. (d) Comparison between reflection from RCWA calculation (black mark) and from multipole moments (grey mark).


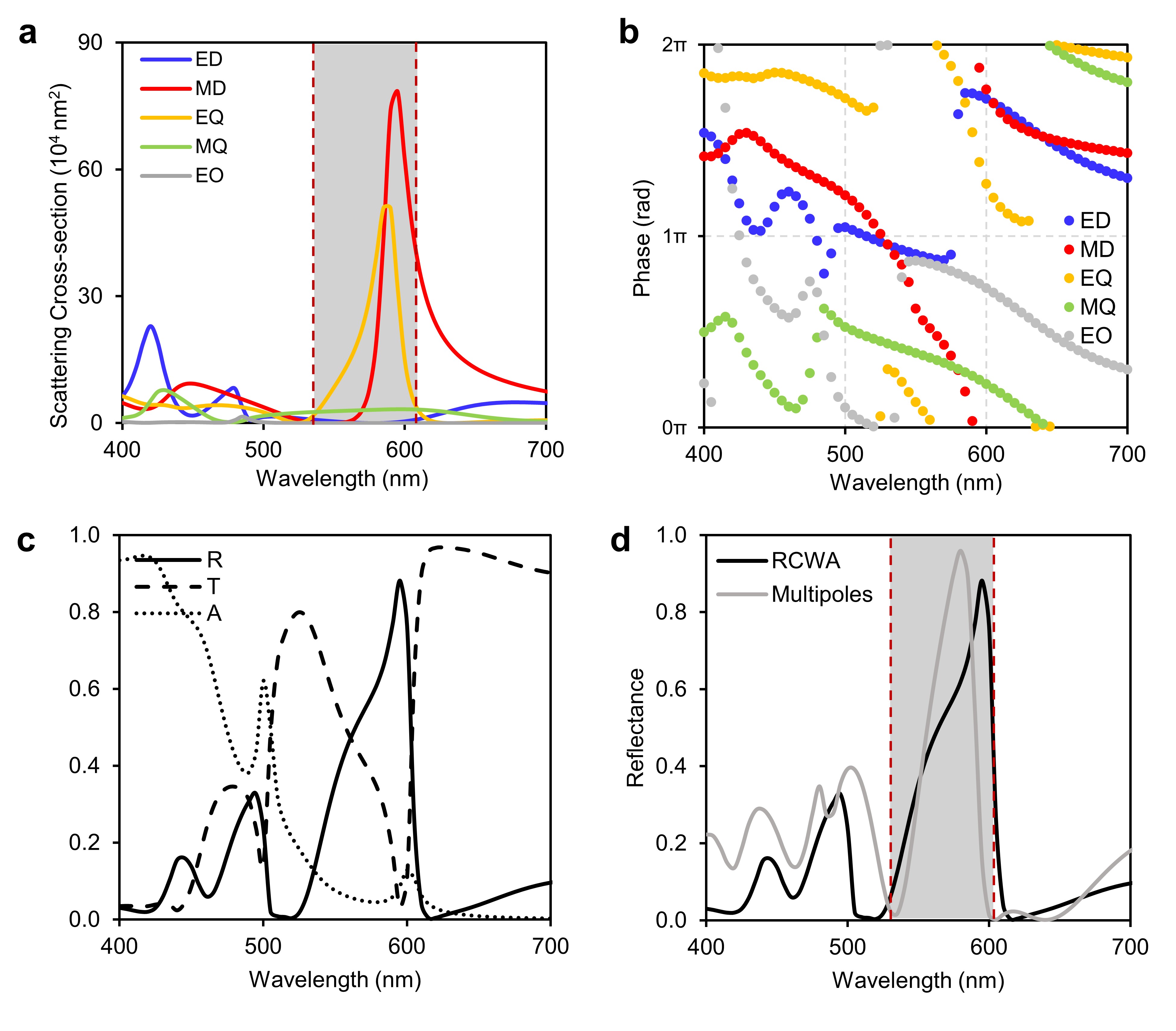


**Supplementary Note 2. Structural colors from an array of randomly rotated meta-atoms.**


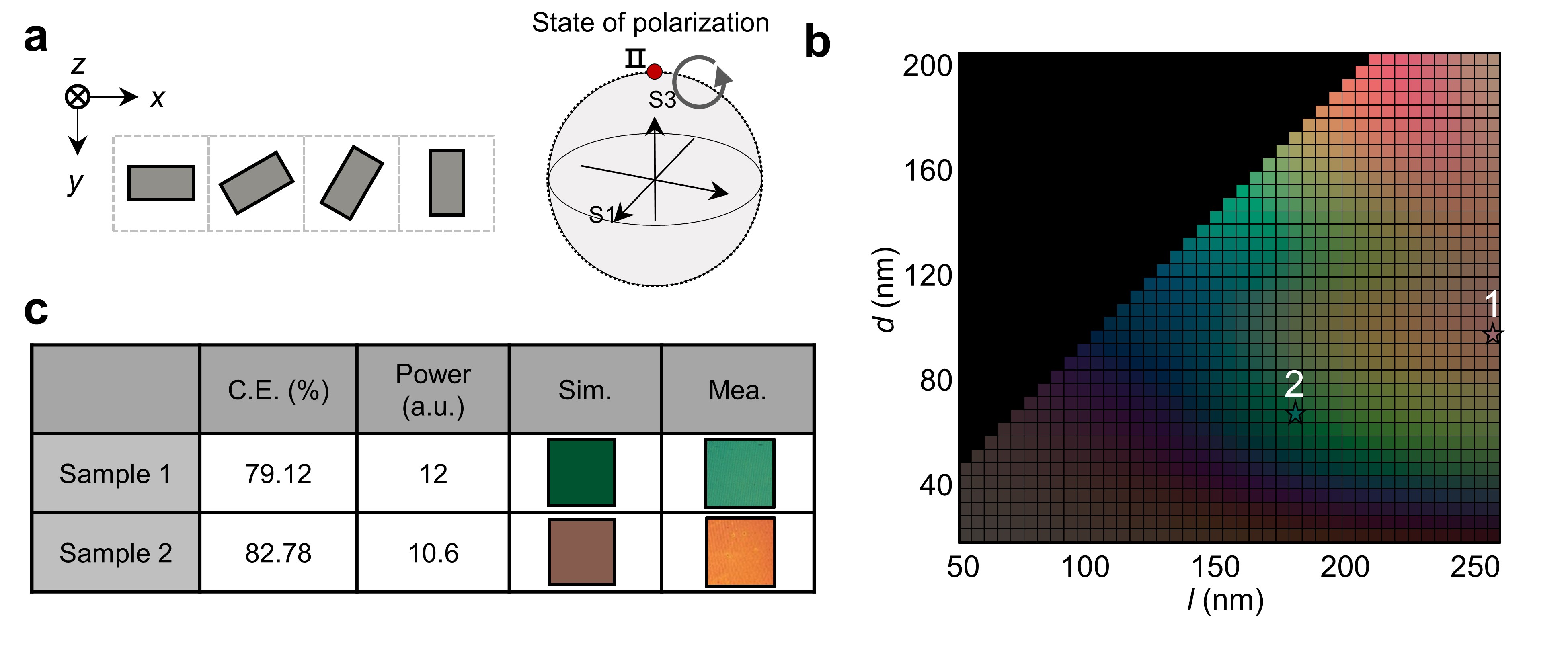


**Supplementary Figure 2. Effects of rotated nanostructures on structural colors.** (a) An unit cell composed of nanostructures used in RCWA simulation. The red dot on the Poincaré sphere represents the polarization state of the incident light. (b) Color palette of the unit cells with the nanostructures which have length l from 50 to 250 nm and width d from 20 to 200 nm. Samples 1 and 2 are denoted by 1 and 2, respectively. (c) Conversion efficiency and colors of Samples 1 and 2. C.E.: calculated conversion efficiency; Power: measured power of beam diffraction caused by rotation of nanostructures in the unit cell. The measured value is normalized by the power of incident light.

The periodic arrangement, i.e. lattice resonance makes a great impact on the spectral modulation by metasurface since its modulation principle is highly related to guided-mode resonance [3]. The geometric perturbation in the array of nanostructure such as rotation, misalignment, or insufficient number of structures in the array may hinder the excitation of the lattice resonance. In this study, the nanostructure in each pixel is rotated to encode phase information retrieved from holograms. The rotation could change the spectral response as compared to when the structure is in array maintaining same rotation angle, e.g. *φ* = 0. To understand this effect, the group of nanostructures of each which unit length and width is *l* and *d*, and rotated counterclockwise direction (Supplementary Figure 2a) is set to unit pixel. The colors of the unit pixels become duller and muddier compared to the case with fixed rotation angle (Fig. 2 and Supplementary Figure 2b). Actually, the result is more likely met to the measured colors of Sample 1 and 2 (Supplementary Figure 2c). This shows that the random rotation in the sample actually impact on its spectral response, which can be one of the reasons of the spectral discrepancy in Fig. 2c.

**Supplementary Note 3. Compensation for light propagation in different waveguide.**


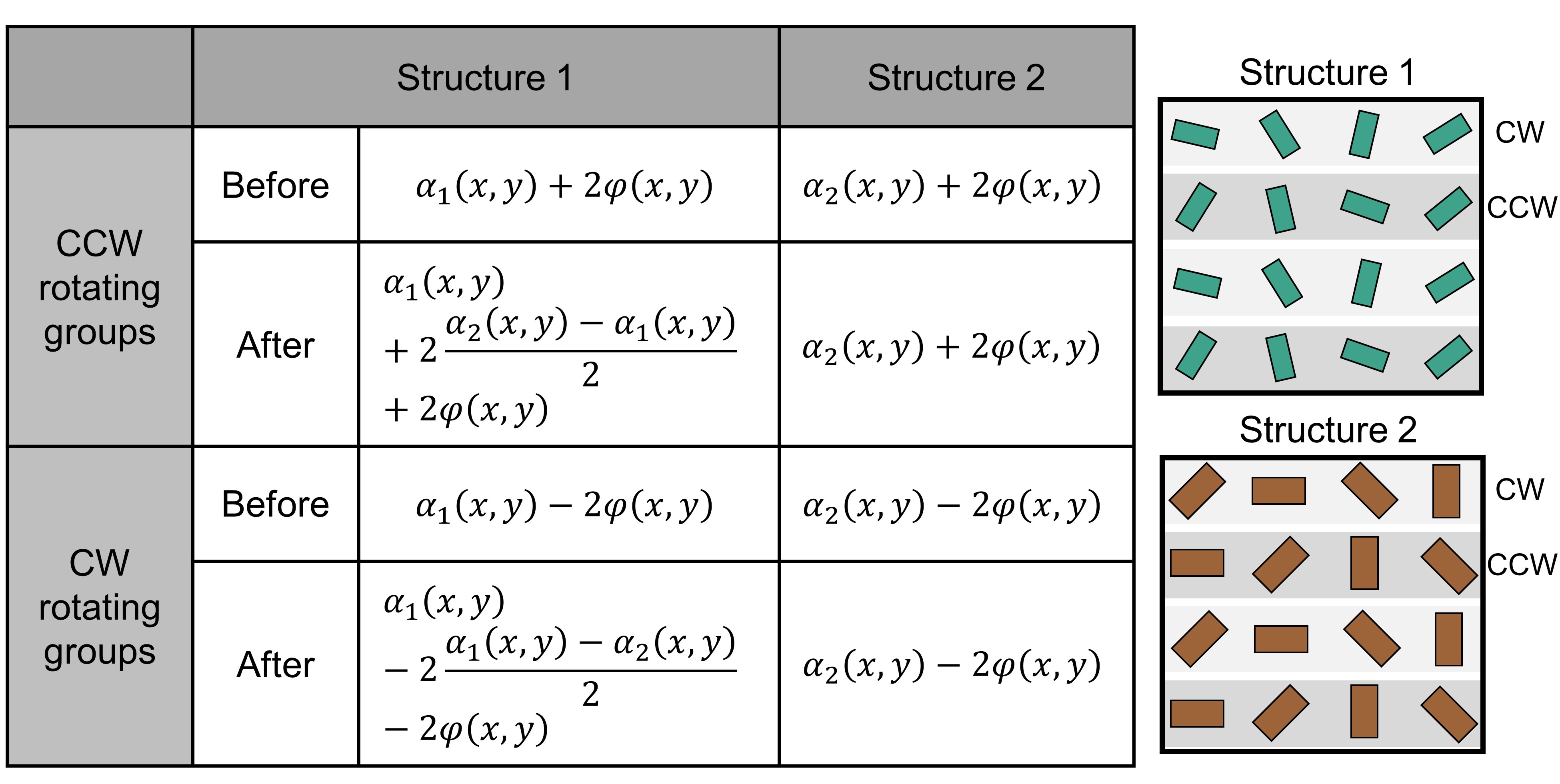


**Supplementary Figure 3. Different propagation phase compensation method for the CCW and CW meta-atom groups.** (Table) Phase information of the structure 1 and 2, where $\alpha\left( x, y \right)$ represents propagation phase, and $\varphi\left( x, y \right)$ represents the rotation angle of the meta-atoms. Meta-atoms of Structure 1 have been additionally rotated for phase compensation. (Figure) The phase of meta-atoms of the Structure 1 has been compensated by an additional CCW (CW) rotation for the meta-atoms at the CCW (CW) meta-atom groups.

Structures 1 and 2 have different spectral reflectance and are used to produce a two-color image. To modulate the phase solely by the in-plane orientation angle of the nanorods, the initial phase compensation is required in advance. Structure 1 and 2 have different geometric parameters, e.g., length and width, where *L_1_* = 250 nm, *W_1_* = 95 nm, *L_2_* = 175 nm and *W_2_* = 65 nm. Corresponding initial phases (propagation phase) are $\alpha_{1}\left( x, y \right)=3.757$ and $\alpha_{2}\left( x, y \right)=1.731$. The initial phase difference $\alpha_{2}\left( x, y \right)-\alpha_{1}\left( x, y \right)$ have been compensated by rotating the structure 1. Corresponding angle is $\frac{\alpha_{2}\left( x, y \right)-\alpha_{1}\left( x, y \right)}{2}$, 58°. According to PB phase, CCW (CW) rotating meta-atom groups convert incident RCP (LCP) to LCP (RCP) with additional phase $2\varphi(x, y)$ ($-2\varphi(x, y)$), where $\varphi(x, y)$ is the in-plane orientation angle. Thus, to compensate the phase difference by additional rotation of the nanorods, the meta-atoms at the CCW (CW) groups should be additionally rotate in CCW (CW) direction. As shown in the figure, the structure 1 meta-atoms of the CCW (CW) groups are rotated 58° in CCW (CW) direction.

**Supplementary Note 4. Refractive index of an optimized hydrogenated amorphous silicon (a-Si:H).**


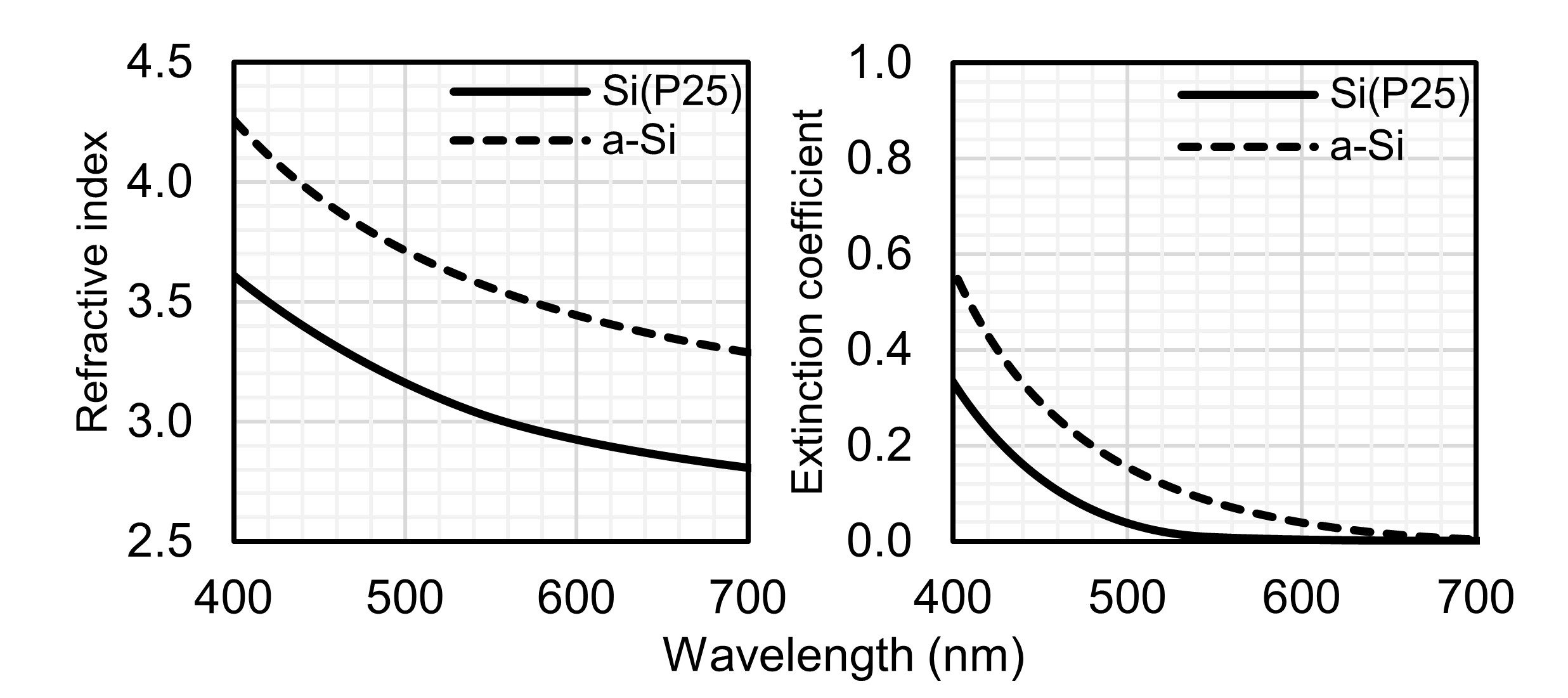


**Supplementary Figure 4. Refractive index of an optimized hydrogenated amorphous silicon.** Real and imaginary refractive index of Si(P25) (solid line) and amorphous silicon (dashed line).

**Supplementary Note 5. Determination of the number of constituent meta-atoms.**

**Supplementary Figure 5. Simulated deflection efficiency of the phase-gradient metasurfaces with different number of meta-atoms.** The far-field intensity and deflected angle of the phase-gradient metasurfaces under circular polarization incidence. The number of meta-atoms that make up meta-atom groups is modulated from 2 to 8 as shown in inset figure. The angle increment of each meta-atom groups is chosen to cover the full phase 0 to 2π.


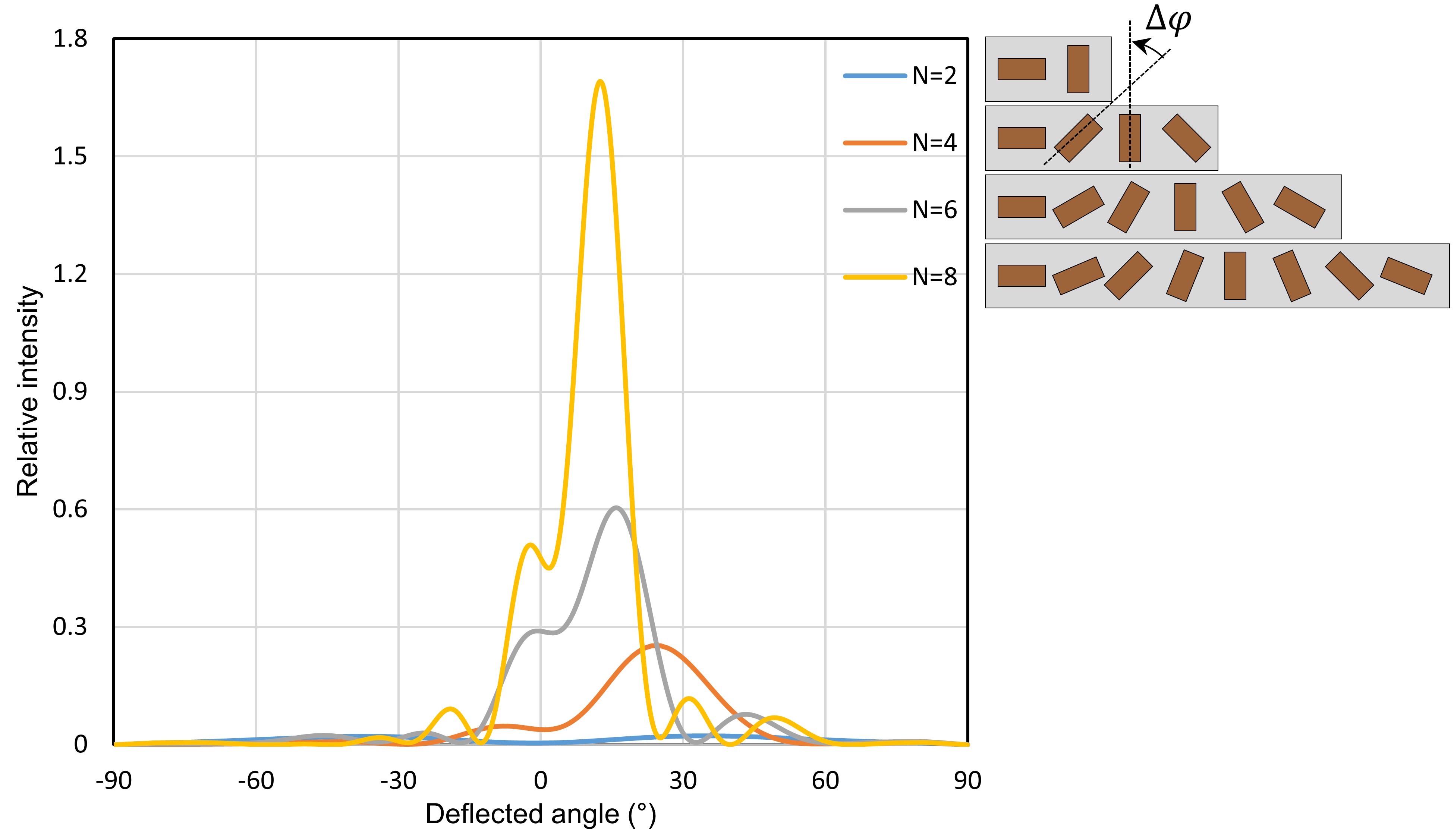


The deflection efficiency (intensity of the deflected light to the intensity of the incident light) of the four types of meta-atom groups have been simulated, where the number of constituent meta-atoms is 2, 4, 6, and 8. The intensity of deflected light is normalized to the number of meta-atoms. The angle increment $\Delta\varphi$ of each meta-atom groups is set to π/2, π/4, π/6 and π/8, covering the full phase 0 to 2π. The corresponding deflection angle is 62.5°, 26.3°, 17.2° and 12.8°. When the number of meta-atoms is eight, the largest deflection efficiency is achieved but the zeroth order beam decreases the clarity of the holographic image, due to the small deflection angle. As a result, we choose the number of constituent meta-atoms as 4 or 6. As shown in Fig. 3c of the main text, the meta-atom groups that make up nine subpixels have four meta-atoms. When the required number of subpixels is decreased, we select the number of constituent meta-atoms as six to balance the deflection efficiency and total footprint of the metasurfaces.

**Supplementary Note 6. Diffraction efficiency measurement and twin-image effect.**

**Supplementary Figure 6. Experimental results of twin-image generation and simulated diffraction efficiency**. (a) Twin-image generation under linearly polarized light illumination. (b) The effect of the incident light polarization to the diffraction efficiency. The diffraction efficiency of the phase-gradient metasurfaces is simulated under circularly and linearly polarized illumination. Here, the phase-gradient metasurface is composed of four meta-atoms covering 2π with four steps.


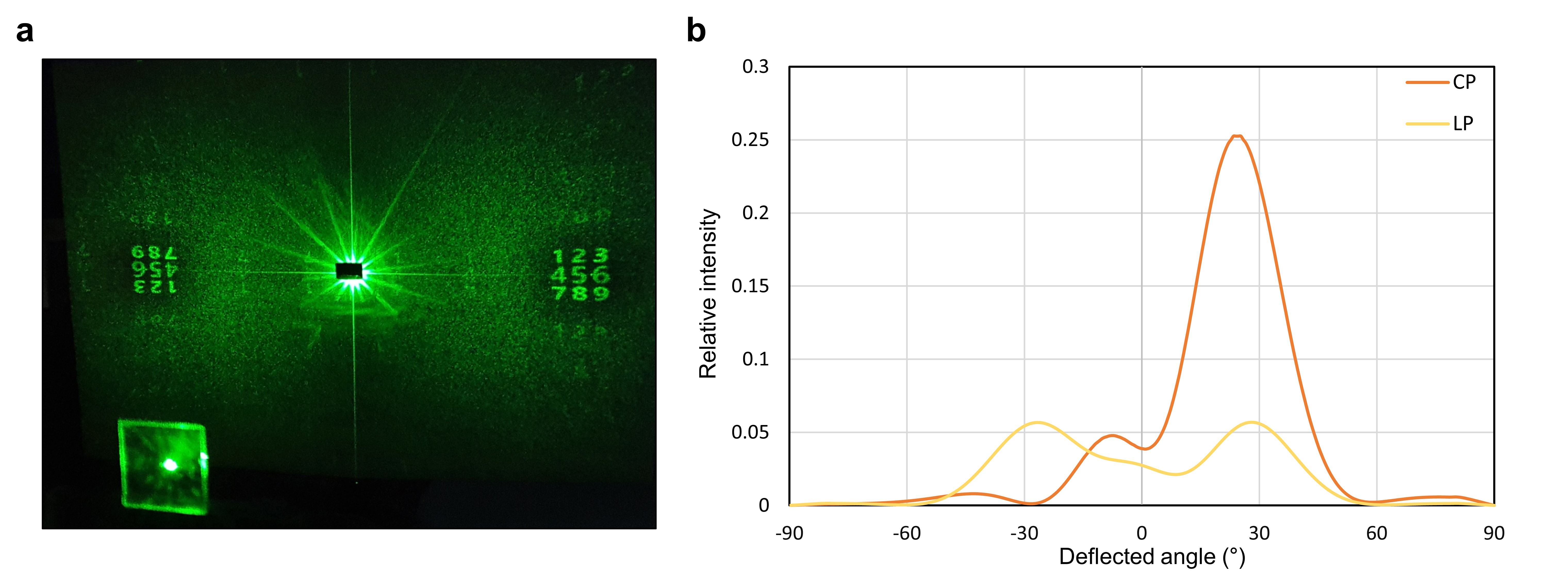


Under linearly polarized light illumination the twin-image is inherently generated and experimentally observed at two opposite angles (Supplementary Figure 6a). The measured absolute efficiency of the generated holographic image is ~4 % for each diffraction angle. As we discussed in the main text, the vectorial metasurfaces are composed of phase-gradient groups with angle increments of $\Delta\varphi$. Under circularly polarized incident light, the cross polarized part of the incident light is deflected at an angle $\theta_{d}=arcsin(\frac{2\Delta\varphi}{k_{0}P})$, where $k_{0}$ denotes the free space wavenumber and *P* denotes the pixel pitch. Here, the phase modulated at each nanorod is $\pm2\varphi\left( x, y \right)$ according to the geometric phase, where the + sign represents RCP and the – sign LCP. Therefore, two different phase fronts, resulting in two opposite deflection angles, are generated under linearly polarized light incident which is a superposition of the two CPs. The effect of incident light on the phase-gradient metasurfaces is matched with numerically plotted intensity over 2π space using finite-difference method in the time domain (FDTD) (Supplementary Figure 6b). Moreover, our vectorial hologram is composed of two types of phase-gradient groups with angle increments of $\Delta\varphi$ and $-\Delta\varphi$, because the vectorial field is generated using two circular polarizations of light with arbitrary amplitudes and phases at the metasurface plane that propagate to the far-field. Therefore, the twin-image is inherently generated from the vectorial metasurfaces composed of two types of phase-gradient meta-atom groups.

**Supplementary Note 7. Jones matrix analysis for output beam intensity profiles**

**
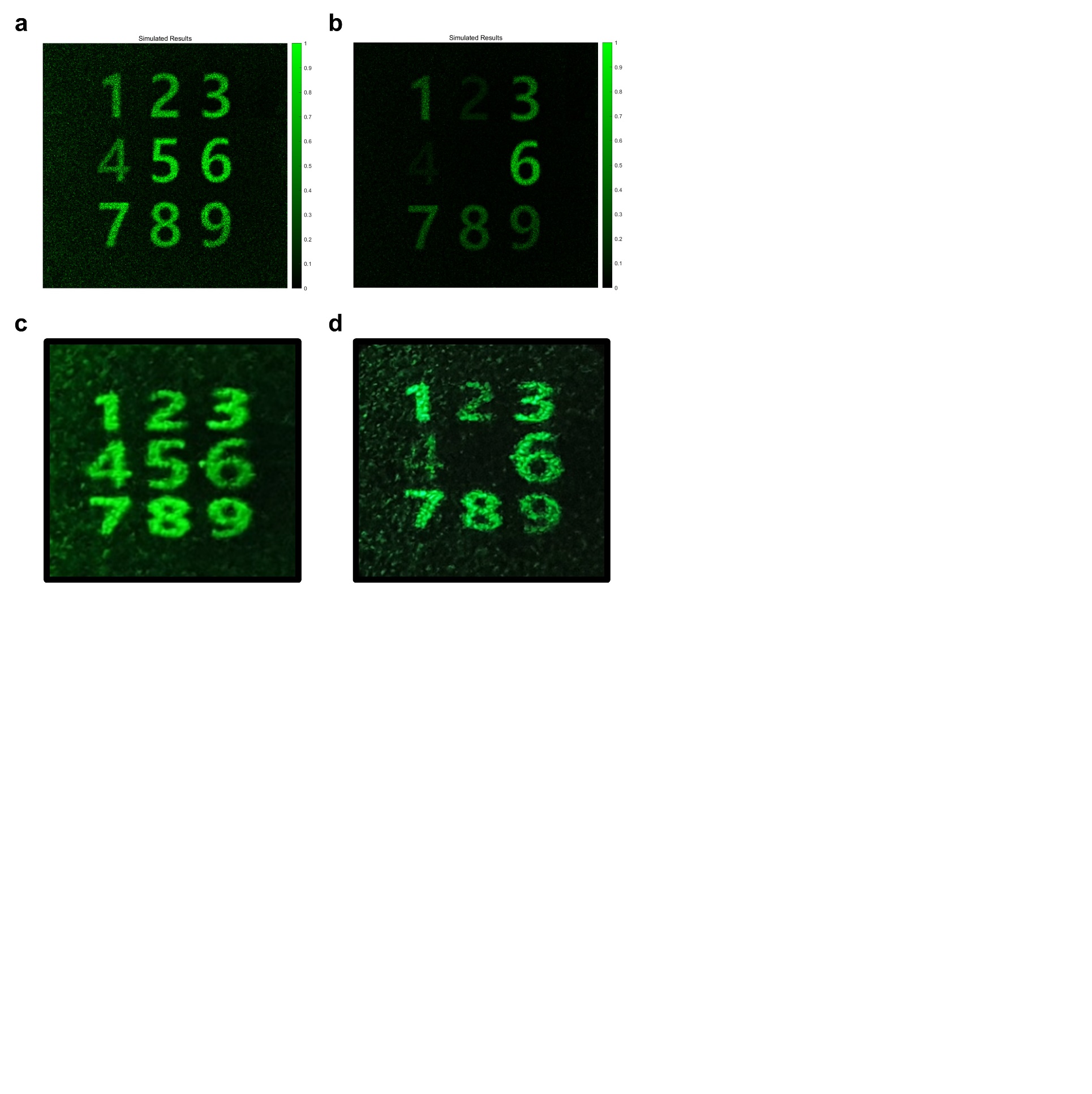
**

**Supplementary Figure 7. Experimental and simulated results of the reconstructed holographic images before and after passing through the linear polarizer**. The (a), (b) simulated and (c), (d) experimental intensity distribution of vectorial holographic images before and after transmitting through the analyzer (45° linear polarizer). Each number has the same polarization state as Fig. 3e.

We characterize the transmission of input waves of multiple polarization states through an optical system represented by the Jones matrix as $\left[ \begin{matrix} {cos}^{2}\theta& sin\theta cos\theta\\ sin\theta cos\theta& {sin}^{2}\theta\end{matrix} \right]$, with a linear polarizer with rotation angle *θ*. In our simulation, the angle *θ* is fixed as 45°. Next, we turn our focus to the Jones vector of input polarization states. A total of nine polarization states are represented by the Jones vectors shown in Supplementary Table 1. The Jones vector of output waves can be obtained from the matrix operation and the intensity is derived as square of the amplitude of electric field. Accordingly, the normalized intensities of the output waves are 0.8, 0.2, 0.8, 0.2, 0, 1, 0.5, 0.5, and 0.5.

**Supplementary Table 1**. Jones vector of input waves.

|  | Polarization state | Circularly polarized bases (RCP : LCP) | Azimuthal angle (rad) | Elliptical angle (rad) | Jones vector |
| --- | --- | --- | --- | --- | --- |
| 1 | Elliptically polarized light | 3:1 | 1.5708 | 0.4643 | $\frac{1}{2\sqrt{5}}\left[ \begin{matrix} 1+3i \\ -3-i \end{matrix} \right]$ |
| 2 | Elliptically polarized light | 3:1 | -1.5708 | 0.4643 | $\frac{1}{2\sqrt{5}}\left[ \begin{matrix} 1-3i \\ 3-i \end{matrix} \right]$ |
| 3 | Elliptically polarized light | 1:3 | 1.5708 | -0.4643 | $\frac{1}{2\sqrt{5}}\left[ \begin{matrix} 1-3i \\ -3+i \end{matrix} \right]$ |
| 4 | Elliptically polarized light | 1:3 | -1.5708 | -0.4643 | $\frac{1}{2\sqrt{5}}\left[ \begin{matrix} 1+3i \\ 3+i \end{matrix} \right]$ |
| 5 | Linearly polarized light | 1:1 | 1.5708 | 0 | $\frac{1}{\sqrt{2}}\left[ \begin{matrix} 1 \\ 1 \end{matrix} \right]$ |
| 6 | Linearly polarized light | 1:1 | -1.5708 | 0 | $\frac{1}{\sqrt{2}}\left[ \begin{matrix} 1 \\ -1 \end{matrix} \right]$ |
| 7 | Circularly polarized light | RCP only | - | 0.7854 | $\frac{1}{\sqrt{2}}\left[ \begin{matrix} 1 \\ i \end{matrix} \right]$ |
| 8 | Circularly polarized light | LCP only | - | -0.7854 | $\frac{1}{\sqrt{2}}\left[ \begin{matrix} 1 \\ -i \end{matrix} \right]$ |
| 9 | Linearly polarized light | 1:1 | 0 | 0 | $\left[ \begin{matrix} 1 \\ 0 \end{matrix} \right]$ |

**Supplementary Note 8. LC cell design**


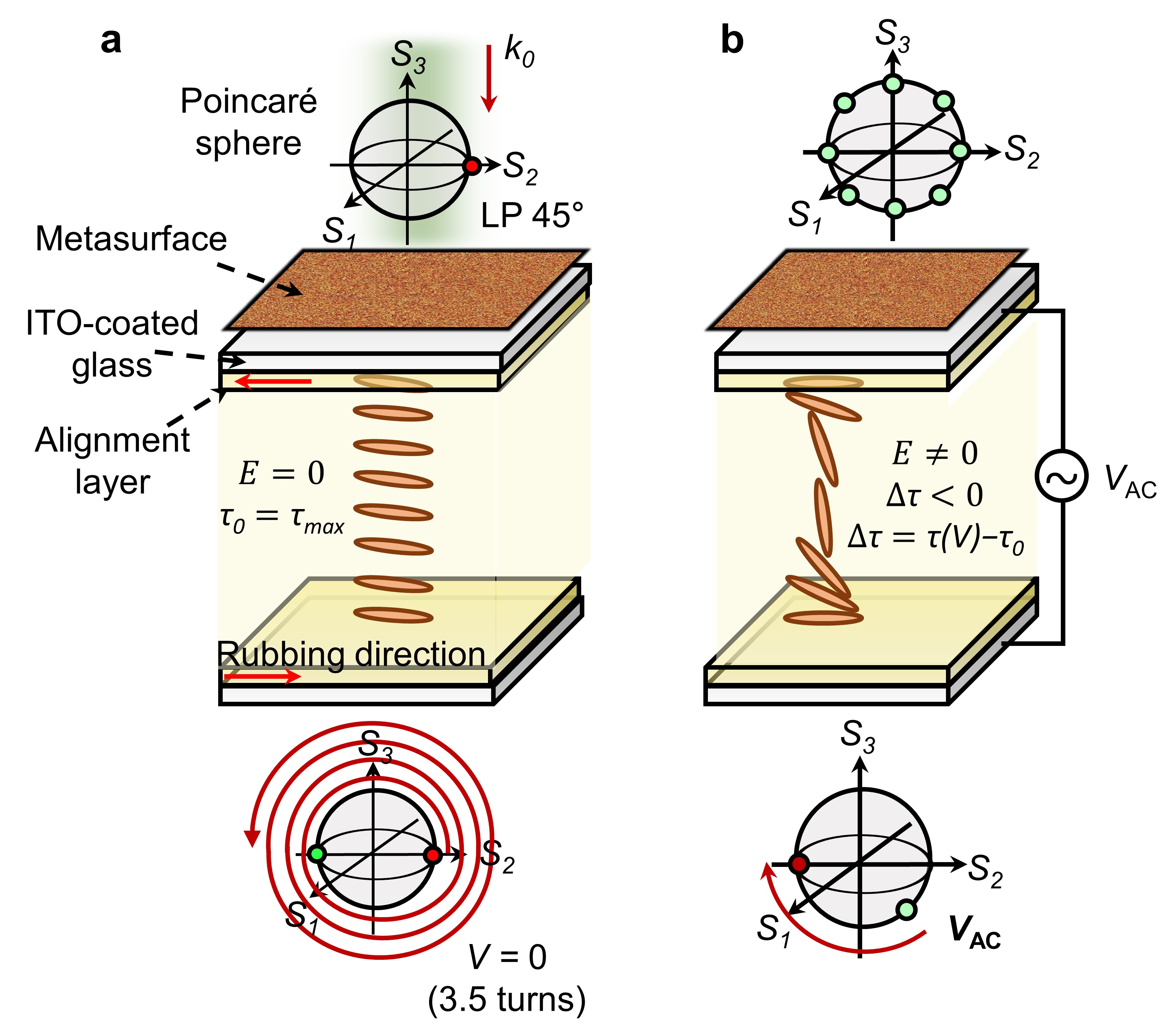


**Supplementary Figure 8. Polarization modulation through a liquid crystal (LC) cell**. The retardation changes are schematically described (a) before and (b) after applying electric bias.

In this work, a specifically designed LC modulator is integrated with the vectorial holographic color prints to provide a much more compact device footprint with precise output beam polarization adjustment. To precisely control the output beam polarization states from the vectorial holographic color prints with a low voltage-bias, a 10 um-thick sandwich-type LC modulator was designed. The retardance and sensitivity of LC modulator to electric bias can be modulated by the thickness of the LC cell or the material of the LC. Here, the LC cell is composed of 5CB LC molecules that have a birefringence Δ*n* = 0.1884 at 532 nm, and according to the rubbing direction of the LCs, the output beam polarization can be modulated along the S2-S3 path on the Poincaré sphere. The initial retardance before applying electric bias was measured to be 22.2 rad, corresponding to 3.5 turns of incident light around the S1 rotation axis on the Poincaré sphere (Supplementary Figure 8a). As the electric bias is applied to the device, the retardance decreases, resulting in a polarization change of the output beam. A specific electric bias that changes each polarization state into a 135° linearly polarized state was experimentally measured and shown in (Supplementary Figure 8b).

Although, we combine the LC modulator and metasurface device for the accurate analysis of vectorial holograms, an optical film-type linear polarizer could also be mounted onto the device to provide a single compact optical platform. In this work, the metasurface device is attached onto the LC modulator where the LC film is confined between two glass substrates. There are a few reasons why the LCs are not in direct contact with the metasurface:

1. The embedded LCs would cause a change to the surrounding medium refractive index, which can reduce diffraction efficiency or induce aberration [4].
2. The LC orientation surrounding the metasurface becomes inhomogeneous resulting in inaccurate phase [5] or spectrum [6] modulation of light.
3. An undesired LC orientation shift affected by the fringe field produced by the metasurfaces will affect the phase or spectrum shift [7].

Since it is important to precisely modulate the retardance in the LC layer in order to sort out the multiple polarization states on the S2-S3 plan of Poincaré sphere, the metasurfaces are fabricated on the LC modulator.

**Supplementary Note 9. Four-color QR code demonstration**


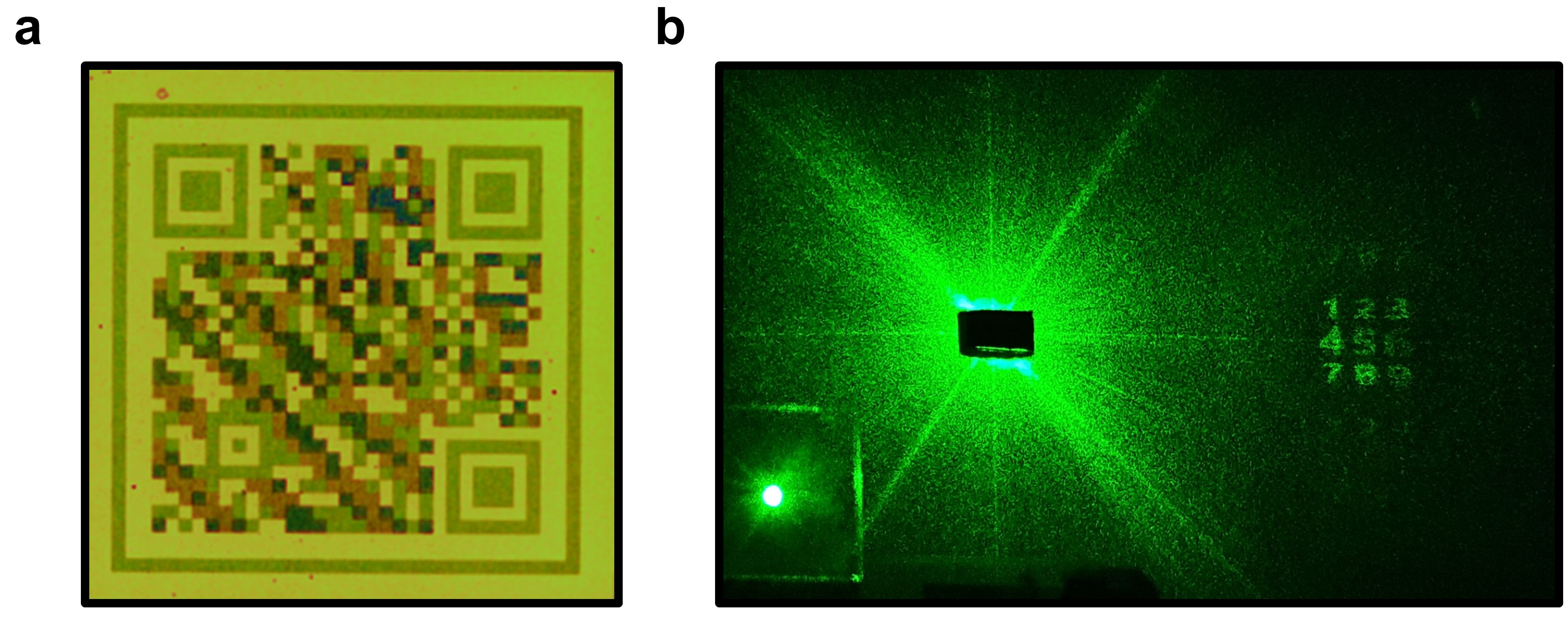


**Supplementary Figure 9. Experimental results of the merged QR codes and far-field vectorial hologram**. (a) Four types of nanostructures are assigned to merge two independent QR codes. (b) Far-field vectorial holographic image from the metasurface that is composed of four different sized nanostructures.

We prepared new sample to highlight the usefulness of the structural coloration for the first key. For the first-level authentication, a two-color QR code is demonstrated using the structural coloration of nanostructures. The vectorial hologram is designed by compensating the different phase retardation from two distinct nanostructures, using the in-plane orientation angle of the nanostructures, to provide second-level authentication. Any arrangement of two types of nanostructures is able to generate dark-bright-pattern that can provide the functionality of a QR code without specific color information. However, one can generate multi-dimensional QR codes that possess higher security levels with multiple encoded information by merging multiple QR codes [8]. It should be noted that four-color information is necessary to merge two independent QR codes, due to the decoding process that requires the color information to split the two merged QR codes. With our design principle of multiplexing colorprints and vectorial holograms, one can increases the density of information by merging QR codes with four different sized nanostructures while maintaining the performance of far-field holographic images. We experimentally observed multi-dimensional QR code (Supplementary Figure 9a) which is generated by merging two independent QR codes and far-field vectorial holographic images (Supplementary Figure 9b).

**References**

1. Mun, J., So, S., Jang, J. & Rho, J. Describing Meta-Atoms Using the Exact Higher-Order Polarizability Tensors. *ACS Photon.* **7**, 1153–1162 (2020).

2. Terekhov, P. D. et al. Multipole analysis of dielectric metasurfaces composed of nonspherical nanoparticles and lattice invisibility effect. *Phys. Rev. B* **99**, 045424 (2019).

3. Jang, J., Badloe, T., Sim, Y. C., Yang, Y., Mun, J., Lee, T., Cho, Y.-H. & Rho, J. Full and gradient structural colouration by lattice amplified gallium nitride Mie-resonators. *Nanoscale* **12**, 21392-21400 (2020).

4. Lininger, A., Zhu, A. Y., Park, J.-S., Palermo, G., Chatterjee, S., Boyd, J., Capasso, F. & Strangi, G. Optical properties of metasurfaces infiltrated with liquid crystals. *Proc. Natl. Acad. Sci. U.S.A.* **117**, 20390-20396 (2020)

5. Li, S.-Q., Xu, X., Veetil, R. M., Valuckas, V., Paniagua-Domínguez, R., Kuznetsov, A. I. Phase-only transmissive spatial light modulator based on tunable dielectric metasurface. *Science* **364**, 1087-1090 (2019).

6. Franklin, D., Chen, Y., Vazquez-Guardado, A., Modak, S., Boroumand, J., Xu, D., Wu, S.-T. & Chanda, D. Polarization-independent actively tunable colour generation on imprinted plasmonic surfaces. *Nat. Commun.* **6**, 7337 (2015)

7. Lee, Y., Park, M.-K., Kim, S., Shin, J. H., Moon, C., Hwang, J. Y., Choi, J.-C., Park, H., Kim, H.-R. & Jang, J. E. Electrical broad tuning of plasmonic color filter employing an asymmetric-lattice nanohole array of metasurface controlled by polarization rotator. *ACS Photon.* **4**, 1954-1966 (2017)

8. You, M., Lin, M., Wang, S., Wang, X., Zhang, Ge., Hong, Y., Dong, Y., Jin, G., Xu, F. Three-dimensional quick response code based on inkjet printing of upconversion fluorescent nanoparticles for drug anti-counterfeiting. *Nanoscale* **8**, 10096-10104 (2016)
