# Supplementary material for: Pixelated bifunctional metasurface-driven dynamic vectorial holographic color prints for photonic security platform
Source: Nat Commun. 2021 Jun 14;12:3614. doi: 10.1038/s41467-021-23814-5 (PMC8203667; doi:10.1038/s41467-021-23814-5)
Supplement: Supplementary file 2 — Description of Additional Supplementary Files [file 41467_2021_23814_MOESM2_ESM.docx]

**Description of Additional Supplementary Files:**

**Supplementary Video 1.**

Demonstration of two-level security platform with vectorial holographic color prints.
